# *Rhus coriaria* L. (Sumac) Evokes Endothelium-Dependent Vasorelaxation of Rat Aorta: Involvement of the cAMP and cGMP Pathways

**DOI:** 10.3389/fphar.2018.00688

**Published:** 2018-06-28

**Authors:** Mohammad A. Anwar, Ali A. Samaha, Safaa Baydoun, Rabah Iratni, Ali H. Eid

**Affiliations:** ^1^Department of Biological and Environmental Sciences, College of Arts and Sciences, Qatar University, Doha, Qatar; ^2^Department of Biomedical Sciences, Lebanese International University, Beirut, Lebanon; ^3^Faculty of Public Health IV, Lebanese University, Beirut, Lebanon; ^4^Research Center for Environment and Development, Beirut Arab University, Beirut, Lebanon; ^5^Department of Biology, College of Science, United Arab Emirates University, Al Ain, United Arab Emirates; ^6^Department of Pharmacology and Toxicology, Faculty of Medicine, American University of Beirut, Beirut, Lebanon

**Keywords:** *Rhus coriaria*, sumac, endothelium-dependent relaxation, phosphoinositide 3-kinase (PI3K), Akt, endothelial nitric oxide synthase (eNOS), adenylyl cyclase (AC), guanylyl cyclase (GC)

## Abstract

*Rhus coriaria* L. (sumac) is widely used in traditional remedies and cuisine of countries of the Mediterranean as well as Central and South-West Asia. Administration of sumac to experimental models and patients with diverse pathological conditions generates multi-faceted propitious effects, including the quality as a vasodilator. Together, the effects are concertedly channeled toward cardiovasobolic protection. However, there is paucity of data on the mechanism of action for sumac’s vasodilatory effect, an attribute which is considered to be advantageous for unhealthy circulatory system. Accordingly, we sought to determine the mechanisms by which sumac elicits its vasorelaxatory effects. We deciphered the signaling networks by application of a range of pharmacological inhibitors, biochemical assays and including the quantification of cyclic nucleotide monophosphates. Herein, we provide evidence that an ethanolic extract of sumac fruit, dose-dependently, relaxes rat isolated aorta. The mechanistic effect is achieved via stimulation of multiple transducers namely PI3-K/Akt, eNOS, NO, guanylyl cyclase, cGMP, and PKG. Interestingly, the arachidonic acid pathway (cyclooxygenases), adenylyl cyclase/cAMP and ATP-dependent potassium channels appear to partake in this sumac-orchestrated attenuation of vascular tone. Clearly, our data support the favorable potential cardio-vasculoprotective action of sumac.

## Introduction

Macro-arterial related dysfunction is the principal contributor to cardiovascular and cerebrovascular diseases (CVDs), such as atherosclerosis (myocardial infarction, stroke, peripheral arterial disease, and renal artery stenosis) and aneurysms. CVDs remain the predominant causes of global morbidity and mortality per annum (Nichols et al., 2014; Writing Group et al., 2016; World Health Organization, 2017). CVDs impart a substantial drain on scarce health-care budgets, worldwide. Much about the etiology of these diseases remains to be understood (White and Olin, 2009; Hopkins, 2013; Davis et al., 2015). However, multiple causative mechanisms have been determined for these highly complex, dynamic arterio-pathological events (Hopkins, 2013; Davis et al., 2015; Bennett et al., 2016; Libby et al., 2016; Writing Group et al., 2016). Despite this immense biomedical knowledge explaining the vascular pathophysiology, together with the significant number of therapeutic armaments prescribed to patients (poly-pharmacy/combinatory medication), there is, nonetheless, an increasing acceptance in clinical practice that CVDs are sub-optimally managed in a significant percentage of patients (August, 2004; Frishman et al., 2009; Wang and Xiong, 2012; Writing Group et al., 2016). Altogether, the aforementioned (lack of response to current medication by patients) alludes to an urgent requirement for novel combinatory therapeutic approaches directed toward multiple patho-targets of CVDs (Bennett et al., 2016; Libby et al., 2016; Writing Group et al., 2016).

Diet, a modifiable lifestyle factor, is well-established as a prominent participant in cardiovascular physiology and pathogenesis (Srinath Reddy and Katan, 2004; Engelfriet et al., 2010; Widmer et al., 2015; Writing Group et al., 2016). Adherence to traditional Mediterranean-type diet is associated with beneficial bio-mechanisms ascertaining to prevention, protection and reduction in adverse cardiovasobolic events, enhanced quality of life, longevity, and consequently lower rates of global mortality (Fung et al., 2009; Bonaccio et al., 2013; Estruch et al., 2013; Rees et al., 2013; Shen et al., 2015; Stewart et al., 2016). Similarly, the Lebanese traditional Mediterranean-related diet has also been reported to be cardiovasculo-protective (Naja et al., 2014; Aljefree and Ahmed, 2015). Moreover, herbs and spices are considered as significant components of the Mediterranean diet (Bower et al., 2016), and are also an essential element of Lebanese-type of Mediterranean cuisine (Jeambey et al., 2009). Contextually, a significant subset of medicinal drugs have botanical origins, which are considered to be a safe and cost-effective alternative to prescription medication (Nearing, 1985; Akerele, 1993; Heber, 2001; Pan et al., 2013; Anwar et al., 2014, 2016; Al Disi et al., 2015). A culinary plant attracting considerable interest, and more germane to the current investigation, is the spice *Rhus coriaria* (RC, a deciduous shrub: Family Anacardiaceae, genus *Rhus*), and commonly referred to as sumac or sumach. The fruits (red berries) of RC remain a component of traditional medicine in countries stretching from the Mediterranean basin (Italy, Turkey, the countries of the Levant) to as far as nations of Central and Western Asia (Shabbir, 2012; Tuttolomondo et al., 2014; Abu-Reidah et al., 2015). Importantly, the fruit of *Rhus coriaria* is endowed with a broad spectrum of phytochemicals such as phenolic compounds, terpenoids, gallic acid, linoleic and oleic acids, kaempferol, quercetin, methyl gallate as well as minerals and vitamins (Kosar et al., 2007; Shabana et al., 2011; Abu-Reidah et al., 2015; Ahangarpour et al., 2017). Some of these phytochemicals are anti-angiogenic (El Hasasna et al., 2016), anti-atherogenic (Zargham and Zargham, 2008), anti-carcinogenic (El Hasasna et al., 2015, 2016), anti-diabetic (Mohammadi et al., 2010), anti-dyslipidemic (Shafiei et al., 2011), antihypertensive (Abu-Reidah et al., 2015; Anwar et al., 2016), anti-oxidative (Candan and Sokmen, 2004), bactericidal (Erturk, 2006), or fungicidal (Erturk, 2006). In addition, extracts of sumac inhibit vascular smooth muscle cell (VSMC) proliferation and migration (Rayne and Mazza, 2007; Zargham and Zargham, 2008; Shabbir, 2012), regulate vascular tone (Beretta et al., 2009), and provide cardioprotection against myocardial ischemia-reperfusion injury (Beretta et al., 2009).

Accumulating evidence show that sumac exhibits multiple, health-promoting cardiovascular effects, including its ability to relax arteries (Beretta et al., 2009). Except for this solitary study, there is dearth of literature on the mechanism of action in sumac-driven arterial relaxation. Hence, the abovementioned investigations provided the impetus for exploring, in detail, the signal transducing pathways in sumac-elicited aortic relaxation. The aorta is a relevant model for investigating pharmacological interventions related to treatment of conduit arterial diseases. In many instances, the results from *in vitro* experiments of aorta treated with drugs have been repeated in small arteries (Faraci and Sigmund, 1999). We hypothesize that *RC-*derived vasorelaxation is mediated through the intracellular signaling systems of guanylyl and adenylyl cyclases (ACs) in aortae of healthy rats, support for which is garnered from aforementioned observations. The results of our investigation represent a significant step toward a better understanding of RC-evoked signal transduction pathways in the vasculature, specifically via participation of cAMP and cGMP routes.

## Materials and Methods

### Chemicals and Drugs

All salts for Krebs–Henseleit buffer were of analytical reagent grade, and together with acetylcholine chloride, 3-isobutyl-1-methylxanthine (IBMX), atropine, indomethacin, norepinephrine, Nω-nitro-L-arginine methyl ester (L-NAME), 1H-[1,2,4]oxadiazolo[4,3-alpha]quinoxalin-1-one (ODQ), pyrilamine, verapamil hydrochloride, glibenclamide, tetraethylammonium (TEA), SQ22536 [9-(tetrahydro-2-furanyl)-9H-purin-6-amine] were purchased from Sigma-Aldrich Co. (St. Louis, MO, United States). LY294002 [2-(4-morpholinyl)-8-phenyl-4H-1-benzopyran-4-one] and Wortmannin were acquired from Bio-Techne (MN, United States) and Alexis Biochemicals (San Diego, CA, United States), respectively.

### Ethics Statement

Institutional consent was acquired for all experimental procedures from the Scientific Committee in the Faculty of Public Health at the Lebanese University [Rhus coriaria’s Vascular Project (Professor Ali Samaha)]. All procedures within this study were performed in strict compliance with the recommendations in the Guide for the Care and Use of Laboratory Animals of the National Institutes of Health.

### Collection and Preparation of an Ethanolic Extract of *Rhus coriaria* Fruits

**FIGURE 1:**
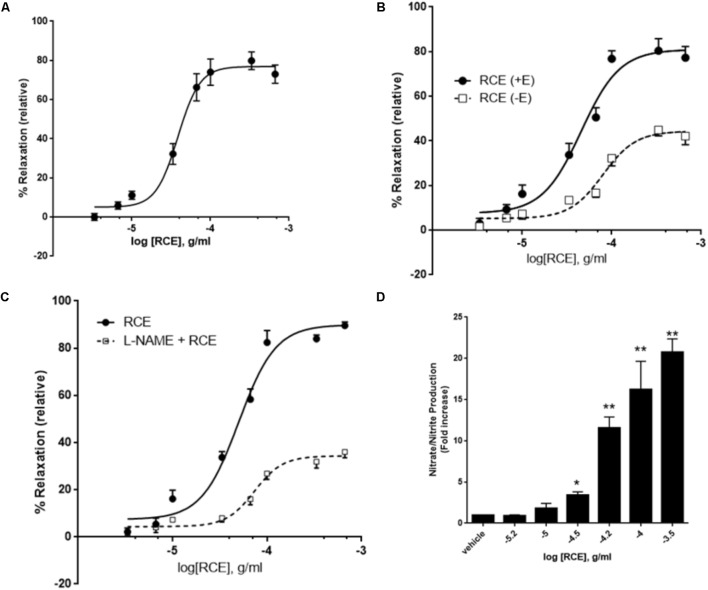
Role of endothelium and NO on RCE-induced relaxation. **(A)** Cumulative dose-dependent relaxation responses to *Rhus coriaria* extract (RCE) on rat isolated aortic rings. Segments of aorta were precontracted with norepinephrine (3 μM). The data expressed are mean ± SEM (*n* = 7). **(B)** Cumulative dose-dependent relaxation curves were generated to sumac (RCE) on norepinephrine-precontracted rat isolated aortic rings, either intact (+E; circles) or devoid of endothelium (–E; squares). The data expressed are mean ± SEM (*n* = 7; *p* < 0.01 for +E versus –E). **(C)** Endothelium-intact aortic rings were subjected to cumulative doses of sumac in the absence (circles) or pre-presence of L-NAME (100 μM; squares). Data represent mean ± SEM (*n* = 7; *p* < 0.01 for RCE versus L-Name plus RCE). **(D)** Endothelium-intact rings were incubated with increasing doses of RCE and levels of NO determined. Each bar displays mean ± SEM, ^∗^*p* < 0.05 and ^∗∗^*p* < 0.01.

The fruits of RC (**Figure 1**) were harvested from the village of Ma’arake (East of Tyre, Lebanon) in the summer of 2009. To stress, RC is not on any endangered or protected species list (local, national, or international); and fruit of the plant is in abundant supply at local markets. Taxonomic authentication was verified by Dr. Ali Al-Khatib, a botanist, at the Lebanese International University, Beirut, Lebanon.

The sumac fruits were separated from the stems, quickly rinsed in distilled water to remove any dust or dirt and air-dried in the dark at room temperature. After grinding the fruit with a pestle in a mortar and throwing the hard seeds, the powder was extracted three times (overnight) in 70% aqueous ethanol and the mixture kept in the dark. The mixture was then filtered through a sintered glass Buchner funnel and the filtrate evaporated to dryness using rotary evaporator at room temperature. The obtained residue was collected and kept at -20°C until further use.

### Animal Care

Male Sprague-Dawley rats (230–290 g; Charles River) were housed in a climatically controlled environment with 12/12 h of light/dark cycle. Animals had access to a standard rat chow and water *ad libitum*. Each experimental protocol consisted of six or more rats per group. The rationale for not using female rats is fundamentally due to the estrous cycle (fluctuation in hormone levels) which will introduce confounding elements into the investigation as the female hormone estrogen is known to affect vasodilatory processes.

### Preparation of Rat Isolated Thoracic Aorta

Prior to experimentation, the rats were humanely killed by an overdose of phenobarbital (50 mg/kg body weight). Immediately, an incision was made through the midline of thoracic-abdominal cavity, the descending thoracic aorta was dissected, and immersed in cold (4°C) modified Krebs-Henseleit buffer [KHB; composition in mM: NaCl (130), Mg_2_SO_4_⋅7H_2_O (1.17), NaHCO_3_ (14.9), KCl (4.7), KH_2_PO_4_ (1.18), CaCl_2_⋅2H_2_O (1.6) and glucose (5.5)], which was pre-equilibrated with 5% CO_2_/95% oxygen. Adhering perivascular adipose and connective tissues were carefully trimmed from the aorta, and ring segments of approximately 3 mm in length were cut for isometric measurements. Each vascular ring was suspended by a combination of stirrup, connected to an isometric force transducer and a stainless steel hook in a pre-warmed (37 ± 1°C) organ bath chamber containing modified KHB (pH 7.4, 37 ± 1°C). The buffer was continuously aerated with 5% CO_2_ in oxygen to maintain the pH of KHB at 7.4. The rings were equilibrated for an hour, subjected to a stepwise increase in tension of 0.5 g every 15 min till a final resting tension of 2 g was attained, subsequent to each incremental rise in tension the rings were washed with fresh buffer (Anwar et al., 2017). Tension changes in aortic ring responses were recorded by a computerized PowerLab data acquisition system (AD Instruments, United Kingdom). Subsequently, the vessel viability was assessed by application of 3 μM NE, followed by two exposures to 80 mM KCl, with thorough washes with modified KHB in between each activation of the aorta. If the tension recordings to the two KCl challenges were similar, the protocol below was followed. In addition, we evaluated endothelial integrity on NE-precontracted aortic segments to 10 μM acetylcholine, and any ring that relaxed less than 85% was discarded.

### Pharmaco-Mechanical Responses

To decipher the signaling routes, each aortic ring was contracted with a suboptimal value of 3 μM NE, on attaining a plateau (approximately 3 min), cumulative dose response curve (CDRC) to sumac was constructed in the absence or presence of various pharmacological inhibitors (Anwar et al., 2013, 2017): L-NAME (100 μM), ODQ (10 μM), indomethacin (10 μM), glibenclamide (10 μM), TEA (100 μM), triciribine (10 μM), wortmannin (100 nM), LY294002 (10 μM), SQ22536 (100 μM), or a vehicle control. In addition to the above set of experimental data, *Rhus coriaria* extract (RCE)-induced arterial relaxation was determined in endothelium-denuded segments (gently rubbing in a to and from movement with a roughened piece of stainless-steel wire, covered tightly in cotton wool, along the intimae of aortic ring-generating less than 15% relaxation with acetylcholine). This was not only to confirm that sumac vasorelaxation is endothelium-dependent, but also to complement the effects of L-NAME on sumac dose-response curves. Penultimately, we tested the possibility of involvement of muscarinic (atropine, 10 μM) and histaminergic (pyrilamine, 10 μM) receptors in RCE-mediated regulation of vascular tone. Lastly, we determined the possibility of calcium channels (verapamil; 1 μM) participating in RCE-evoked aortic relaxation.

### Quantification of Nitrate and Nitrite

Nitric oxide (NO) release was assessed by application of Griess reaction, as described previously (Anwar et al., 2017). The manufacturer’s protocol was followed for measurement of total nitrate and nitrite content (NO_x_; a measure of NO released) using a colorimetric assay kit (Cayman Chemical Company, Ann Arbor, MI, United States). Briefly, aortic rings were incubated with increasing concentrations of sumac for 30 min at 37°C to stimulate the release of NO, which is rapidly converted to nitrate. This metabolite was enzymatically reduced to nitrite by nitrate reductase. Aortae were homogenized, centrifuged, and the supernatant mixed with Griess reagent for 10 min at room temperature. During this incubation period, the nitrite was converted to a deep purple azo-compound, the absorbance of which was read at 540 nm. The total content of nitrite was calculated from a nitrite standard curve, and normalized to the protein content (Bradford’s method) of aortic segments.

### Quantification of Cyclic Nucleotide Monophosphates in Rat Thoracic Aortic Tissue

Both cGMP and cAMP were quantitated in aortic rings exposed to sumac, as described previously (Anwar et al., 2017). Briefly, pre-equilibrated rings were treated with IBMX for 30 min before addition of NE. After equilibrating the rings for an additional 30 min, RC was added. The reaction was stopped by freezing the tissues in liquid nitrogen. After homogenization, samples were centrifuged at 10,000 × *g* for 10 min and the supernatant was extracted five times with water-saturated diethyl ether. The cGMP and protein content in the extract were determined by a specific immunoassay [Amersham Biosciences (now GE Healthcare Life Sciences), United States] and by the use of Bradford’s method, respectively. Results are expressed as pmoles of cGMP per milligram of protein. To measure cAMP levels, frozen aortae were homogenized in 0.1 mol/l hydrochloric acid and centrifuged at 1,000 × *g* for 15 min at room temperature. The supernatant were used in the assay as per the manufacturer’s instructions (CA200, Sigma-Aldrich, United States).

### Statistical Analysis

All results are expressed as mean ± standard error of mean (SEM), and n is the number of animals used per group or where applicable refers to number of experiments. A single cumulative dose dependent curve was performed per segment. All relaxant responses to sumac are expressed as a percentage decrease in the tone generated by norepinephrine (3 μM). Data were analyzed with GraphPad Prism software (Version 6, GraphPad Software, Inc., La Jolla, CA, United States) using a four-parameter logistic curve fitted to the Hill equation (with variable slope). The dose of sumac causing 50% relaxation of NE contraction is presented as pD_50_ (g/ml) value (pD_50_ = -log ED_50_), whereas *E*_max_ (%) is the maximal relaxation value obtained. It is important to note here that the rationale for using dose instead of concentration arises from the fact that we do not know the precise concentration of pharmacologically active constituents of RCE. Statistical analysis was performed using two-tailed Student’s *t*-test when comparing two groups. Inter- and intra-group variations were determined by use of analysis of variance (ANOVA), one-way for multiple comparisons or two-way repeated measures, with Sidak’s *post hoc* test. Differences between groups were considered as significant when a *P*-value was set at 0.05.

## Results

### Proof of Concept

Initially, we assessed the exposure of rat isolated aortic rings (endothelium-intact) to cumulative doses of RCE (0.3 μg/ml – 1.0 mg/ml, at half-log doses). **Figure 1A** illustrates a proof of concept for RCE-induced, dose-dependent, relaxation of rat isolated aortic rings pre-contracted with norepinephrine (3 μM). The pED_50_ of sumac’s effect is 4.41 ± 0.04 g/ml with a 95% confidence interval of 4.94–4.33 g/ml), and a corresponding *E*_max_ of 77 ± 3%.

### Involvement of Endothelium, Nitric Oxide, and Soluble Guanylyl Cyclase in RCE-Induced Vasorelaxation

We next wished to determine the role of endothelium in the RCE-induced relaxation of aortic rings in order to gain a better mechanistic understanding that underpins this effect. Indeed, the RCE-induced maximal arterio-relaxant response (*E*_max_) was dramatically decreased with endothelial denudation, and no alteration in pED_50_ values was observed (**Figure 1B**). pED_50_ values were 4.34 ± 0.05 g/ml with 95% confidence interval of 4.45–4.24 g/ml for the endothelium-intact rings versus 4.09 ± 0.05 g/ml and a 95% confidence interval: 4.19–4.00 g/ml for the denuded ones (*p* < 0.01). Similarly, *E*_max_ was 81 ± 4 versus 44 ± 2% for endothelium-intact or denuded rings, respectively (*p* < 0.01). These results underscore the importance of the endothelial layer in RCE-induced vasorelaxation.

Next, we determined the involvement of eNOS in the observed relaxation phenomenon. In the presence of L-NAME, an arginine analog that inhibits eNOS, the RCE-evoked maximal relaxation was robustly dampened (**Figure 1C**). In control rings treated only with sumac (RCE), pED_50_ was 4.34 ± 0.04 g/ml with a 95% confidence interval of 4.41–4.27 g/ml. However, in the L-NAME-treated rings (L-NAME + RCE), pED_50_ was 4.13 ± 0.04 g/ml with 95% confidence interval of 4.22–4.05 g/ml (*p* < 0.05). Moreover, *E*_max_ value was 89 ± 3 prior to L-NAME exposure and 34 ± 2% in the presence of L-NAME (*p* < 0.001). These results were further confirmed by assays that determine NOS activity, measured by conversion of L-arginine to L-citrulline. Our findings also show that RCE significantly increased NOS activity compared to vehicle treatment (data not shown).

To confirm the involvement of NO, levels of this gasotransmitter were measured in RCE-treated rings. **Figure 1D** shows a significant and dose-dependent increase in the production of nitrate/nitrite, indicative of increased NO production. Moreover, pre-treatment with L-NAME inhibited RCE-induced increase in NO (data now shown). As such, and in combination with the above results, it appears that sumac elicits its effects via increasing NO levels.

**FIGURE 2:**
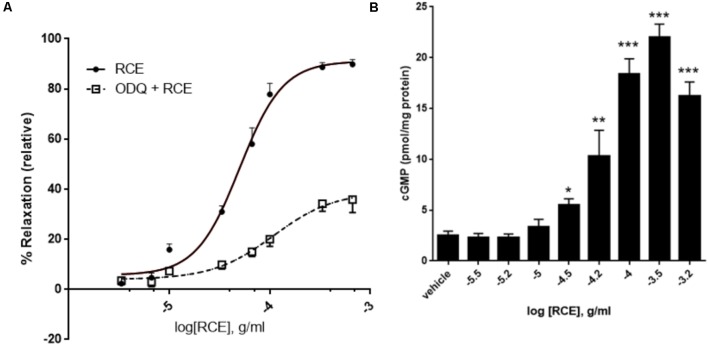
Role of guanylyl cyclase and cGMP in RCE-induced relaxation. **(A)** Endothelium-intact aortic rings were subjected to cumulative doses of sumac without (circles; RCE) or with pre-incubation with ODQ (1 μM; squares; ODQ + RCE). Data represent mean ± SEM (*n* = 7; *p* < 0.01 for RCE versus ODQ + RCE). **(B)** Accumulation of cGMP in aortic rings in the absence or presence of increasing doses of RCE. Each bar represents mean ± SEM (^∗^*p* < 0.05, ^∗∗^*p* < 0.001, and ^∗∗∗^*p* < 0.0001).

Next, we sought to investigate if soluble guanylyl cyclase (sGC) is involved. We therefore analyzed the modifying effect of ODQ (a selective inhibitor of sGC) on RCE-elicited CDRC. **Figure 2A** shows that in rings treated with sumac alone (RCE), pED_50_ was 4.30 ± 0.03 g/ml with a 95% confidence interval of 4.37–4.23 g/ml, whereas in the presence of ODQ (ODQ + RCE), pED_50_ was 3.96 ± 0.14 g/ml with a 95% confidence interval of 4.25–3.68 g/ml (*p* < 0.05). However, *E*_max_ of rings treated with sumac alone (RCE) was 91 ± 3%, while that of ODQ treated rings (ODQ + RCE) was significantly reduced to 39 ± 4% (*p* < 0.001). There is a striking reduction in maximal relaxation response, but no change in pED_50_ values between the two curves.

**FIGURE 3:**
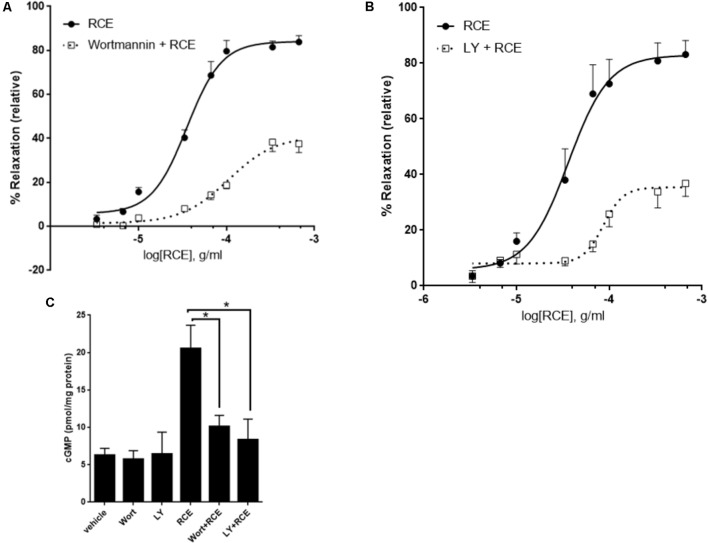
PI3-K/Akt axis is actively involved in RCE-driven relaxation of rat aorta. Endothelium-intact rings were exposed to cumulative doses of sumac in the absence (circles) or prior presence of **(A)** Wortmannin (0.1 μM; squares) or **(B)** LY294002 (10 μM; squares). Data represents mean ± SEM (*n* = 7; *p* < 0.01 for RCE versus either Wortmannin + RCE or LY294002 + RCE). **(C)** Rings were incubated without or with RCE after having been pre-treated without (vehicle) or with wortmannin (0.1 μM) or LY294002 (10 μM) for 30 min. cGMP levels were then assayed. *n* = 7; ^∗^*p* < 0.01.

**FIGURE 4:**
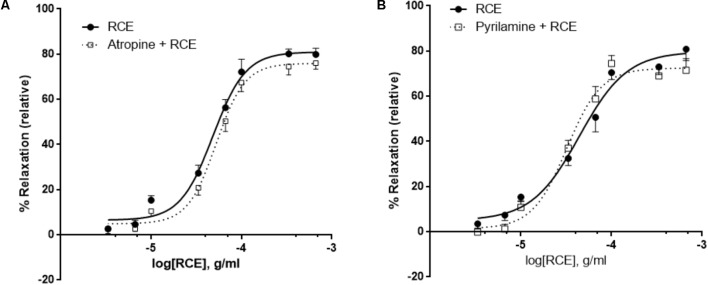
Antagonism of histaminic or muscarinic receptors does not affect RCE-induced relaxation. Endothelium-intact rings were incubated with cumulative doses of sumac in the absence (RCE; circles) or presence of **(A)** 10 μM of atropine (atropine + RCE; squares) or **(B)** 10 μM of pyrilamine (pyrilamine + RCE; squares). Data represent mean ± SEM (*n* = 7 for pyrilamine or atropine). *p*> 0.05 for sumac alone versus either pyrilamine + RCE or atropine + RCE.

When NO docks on its binding site within sGC, cGMP is produced by the cyclization of GTP. The release of cGMP progressively rises with increasing doses of RCE (**Figure 2B**; *p* < 0.001). The production of cGMP in response to sumac follows a sigmoidal trend, reflecting the CDRCs of **Figures 2**, **3A,B**, **4A**. This is further confirmed by our observation that pre-treatment with L-NAME or ODQ significantly diminishes RCE-induced cGMP production (data now shown).

### Inhibition of Phosphoinositide-3 Kinase (PI3K) and Akt in RCE-Generated Vasorelaxation

The role of the PI3K-Akt signaling in endothelium-dependent vasodilation is overwhelmingly documented. The key enzyme, eNOS, is a direct downstream target of the kinase Akt. To determine if the PI3K-Akt pathway is involved, we examined the effect of PI3K and Akt inhibitors on RCE-evoked aortic relaxation. Two structurally diverse blockers, wortmannin and LY294002 that effectively inhibit PI3K signaling, significantly reduced sumac’s vasodilatory effects. **Figure 3A** shows that in the absence of any inhibitor (RCE), pED_50_ is 4.45 ± 0.04 g/ml with a 95% confidence interval of 4.53–4.37 g/ml; however, in wortmannin-pretreated rings (Wort + RCE), pED_50_ is 3.98 ± 0.09 g/ml with a 95% confidence interval: 4.16–3.79 g/ml (*p* < 0.05). Similarly, *E*_max_ was 84 ± 3 or 41 ± 4% (*p* < 0.001) for rings treated with sumac in the absence (RCE) or presence of wortmannin (Wort + RCE), respectively. Similar results were obtained with LY294002 (**Figure 3B**). Indeed, pED_50_ was 4.44 ± 0.09 g/ml with a 95% confidence interval of 4.62–4.27 g/ml for rings treated with sumac alone (RCE) versus 4.06 ± 0.06 g/ml with a 95% confidence interval of 4.18–3.95 g/ml for rings pretreated with LY294002 (LY + RCE) (*p* < 0.01). Likewise, *E*_max_ was 83 ± 5 or 35 ± 3% (*p* < 0.001) for rings pretreated without or with LY294002, respectively. Furthermore, sumac failed to induce significant vasodilation in vessels pre-treated with triciribine (10 μM), a selective inhibitor of Akt but not PI3K (data not shown), clearly implicating the Akt pathway in the observed vasorelaxation. Importantly, levels of cGMP were significantly diminished (*p* < 0.01) in RCE-treated rings that were pre-exposed to wortmannin or LY294002 (**Figure 3C**). It is worth mentioning that pre-treatment with both LY294002 and L-NAME did not show any additive effect over either drug alone (**Supplementary Figure S1**), suggesting that effects elicited by Akt and eNOS are likely one rather than two separate pathways.

### RCE-Elicited Vasorelaxation Is Independent of Muscarinic and Histaminergic Receptors

It has been established that activation of endothelial muscarinic M3 receptors leads to the release of NO (Attina et al., 2008). In our study, the addition of a non-selective muscarinic receptor blocker (atropine) showed no change in RCE-derived relaxation when compared to controls (**Figure 4A**). pED_50_ values were 4.32 ± 0.03 g/ml with 95% confidence interval of 4.38–4.25 g/ml or 4.28 ± 0.03 g/ml with a 95% confidence interval of 4.34–4.22 g/ml (*p* > 0.05) for rings treated with sumac in the absence (RCE) or presence (atropine + RCE) of atropine, respectively. Values for *E*_max_ were 81 ± 2 or 76 ± 2% (*p* > 0.05) in the absence or presence of atropine, respectively. Likewise, aortic relaxation by sumac was unaffected by pre-exposure to pyrilamine (also known as mepyramine, histamine H1 receptor antagonist) (**Figure 4B**). pED_50_ was 4.35 ± 0.06 g/ml with 95% confidence interval of 4.47–4.24 g/ml or 4.49 ± 0.05 g/ml with 95% confidence interval of 4.59–4.39 g/ml) (*p* > 0.05) and *E*_max_ values were 80 ± 4 or 72 ± 3%) (*p* > 0.05) in the absence (RCE) or presence of pyrilamine (pyrilamine + RCE), respectively.

### Effect of Indomethacin on RCE-Dependent Aortic Relaxation

**FIGURE 5:**
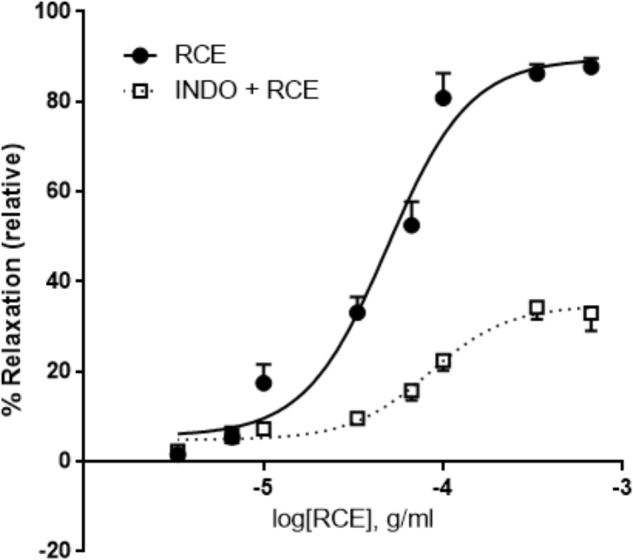
Inhibition of cyclooxygenases attenuates RCE-induced relaxation of aortic rings. Endothelium-intact rings were incubated with cumulative doses of sumac in the absence (RCE; circles) or presence of indomethacin (10 μM; INDO + RCE; squares). Data represent mean ± SEM (*n* = 7; *p* < 0.01).

Pre-incubation with indomethacin, a non-selective cyclooxygenase (COX) inhibitor, (INDO + RCE) shows striking mitigation of *E*_max_ parameter for RCE-generated reduction in aortic tone (**Figure 5**) compared to that produced in the presence of RCE alone (90 ± 3 vs. 35 ± 2%) (*p* < 0.01). Moreover, pED_50_ values were significantly different as well: 4.31 ± 0.04 g/ml (95% confidence interval: 4.40–4.22 g/ml) vs. 4.08 ± 0.06 g/ml (95% confidence interval: 4.21–3.95 g/ml) (*p* < 0.05) in the absence (RCE) or presence of indomethacin (INDO + RCE), respectively. Together, this suppression of the vasorelaxatory response suggests the involvement of prostanoid(s) in RCE-induced arterial relaxation.

### Role of Potassium Channels in RCE-Induced Relaxation

**FIGURE 6:**
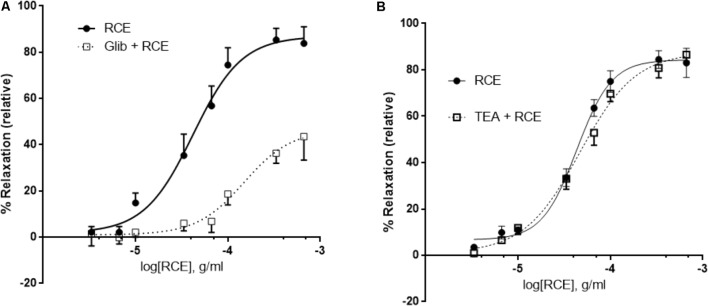
RCE-induced relaxation of aortic rings is mediated by ATP-gated potassium channel activity. Endothelium-intact rings were incubated with cumulative doses of sumac in the absence (RCE; circles) or presence of **(A)** 10 μM of glibenclamide, an ATP-sensitive potassium channels blocker (Glib + RCE; squares) or **(B)** tetraethylammonium (100 μM), a non-selective inhibitor of Ca^2+^-sensitive potassium channels (TEA + RCE; squares). Data represent mean ± SEM (*n* = 7 for glibenclamide or TEA experiments). *p* < 0.05 for RCE alone versus Glib + RCE and *p*> 0.05 for RCE only against RCE + TEA.

Pre-incubation with glibenclamide, a blocker of ATP-gated potassium channels (K_ATP_ channels) robustly attenuated RCE-induced aortic relaxation (**Figure 6A**). pED_50_ values in the absence (RCE) or presence of glibenclamide (Glib + RCE) were, respectively, 4.38 ± 0.04 g/ml (95% confidence interval: 4.45–4.30 g/ml) or 3.82 ± 0.09 g/ml (95% confidence interval: 4.01–3.64 g/ml); (*p* < 0.01). *E*_max_ was 87 ± 3 or 47 ± 5% in the absence (RCE) or presence of glibenclamide (Glib + RCE), respectively; (*p* < 0.01). In contrast, exposure to TEA (non-selective antagonist of Ca^2+^-sensitive potassium channels, K_Ca_ channels) generated CDRCs similar to those produced without the K_Ca_ channels blocker (**Figure 6B**). pED_50_ was 4.36 ± 0.04 g/ml (95% confidence interval: 4.44–4.29 g/ml) or 4.33 ± 0.14 g/ml (95% confidence interval: 4.44–4.23 g/ml), (*p* > 0.05); and *E*_max_ 84 ± 3 or 88 ± 4% (*p* > 0.05) in the absence (RCE) or presence of TEA (TEA + RCE), respectively. Treatment with iberiotoxin elicited similar effects to TEA (data not shown). These data clearly illustrate that sumac relaxes aorta by activation of K_ATP_ channels, independently of K_Ca_ channels.

### Effect of Adenylyl Cyclase Blockade on RCE-Sensitive Arterial Relaxation

**FIGURE 7:**
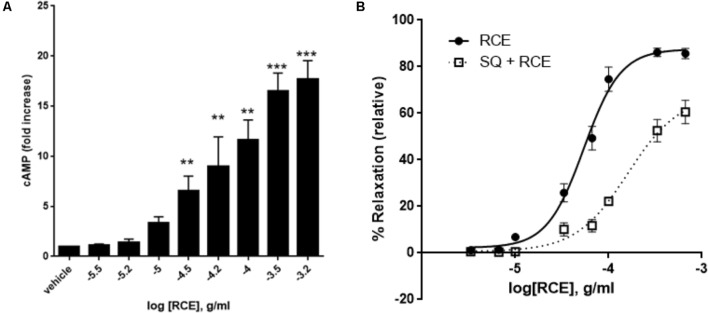
Role of cAMP and adenylyl cyclase in RCE-elicited aortic relaxation. **(A)** Accumulation of cAMP in aortic rings in the absence or presence of increasing doses of RCE. Each bar represents mean ± SEM (^∗∗^*p* < 0.001 and ^∗∗∗^*p* < 0.0001). **(B)** Endothelium-intact rings were treated with cumulative doses of sumac in the absence (RCE; circles) or presence (SQ + RCE; squares) of SQ22536 (100 μM). Values are mean ± SEM, *n* = 7; *p* < 0.05.

We first tested the effect of RCE on cAMP accumulation. Indeed, RCE causes a significant and concentration-dependent increase in levels of cAMP (**Figure 7A**). The contribution of this cAMP to RCE-induced relaxation becomes evident in the presence of the AC inhibitor SQ 22,536. Indeed, pre-exposure to SQ22536 reduced the sensitivity by causing a shift to the right (**Figure 7B**). pED_50_ is 4.27 ± 0.03 g/ml (95% confidence interval: 4.32–4.21 g/ml, RCE) vs. 3.79 ± 0.10 g/ml (95% confidence interval: 4.00–3.59 g/ml, RCE + SQ22536); *p* < 0.05. Similarly, SQ22536 attenuated the maximal relaxation: *E*_max_: 88 ± 3 (RCE) vs. 68 ± 8 (RCE + SQ22536)%; *p* < 0.05. These results imply that AC is recruited in RCE-induced relaxation. Furthermore, pre-treatment with both LY294002 and SQ22536 did not show any additive effect over either drug alone (**Supplementary Figure S2**). This suggests that cAMP and Akt converge onto one pathway to mediate the observed RCE-induced relaxation.

### Effect of Verapamil (V) on RCE-Evoked Relaxation

**FIGURE 8:**
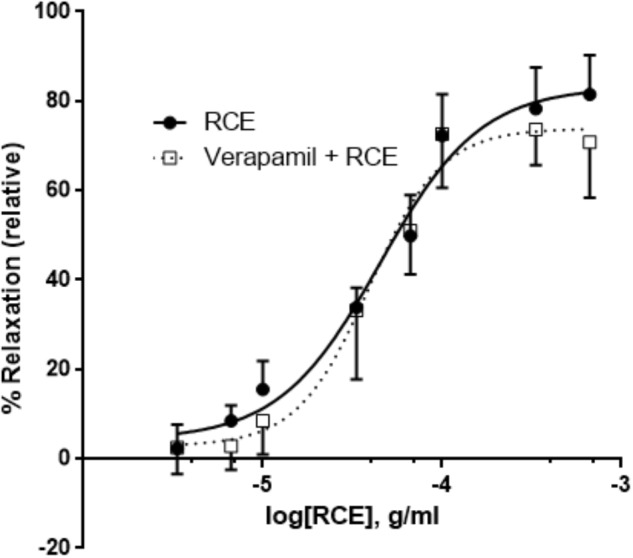
Verapamil plays no role in RCE-induced relaxation of rat aortic segments. Endothelium-intact rings were incubated with cumulative doses of sumac in the absence (RCE; circles) or presence of verapamil (a L-type calcium channel blocker, 1 μM; verap + RCE; squares). Data represent mean ± SEM (*n* = 6; *p*> 0.05).

Pretreatment of aortic rings with verapamil hydrochloride (L-type voltage operated calcium channel blocker) produced no changes in pED_50_ [4.35 ± 0.05 g/ml (95% confidence interval: 4.44–4.25 g/ml, RCE) vs. 4.41 ± 0.05 g/ml (95% confidence interval: 4.51–4.30 g/ml, RCE + V); *p* > 0.05] nor did it affect maximal relaxant response elicited by sumac: *E*_max_ (83 ± 3 (RCE) vs. 74 ± 3 (RCE + V)%; *p* > 0.05] elicited by sumac (**Figure 8**).

## Discussion

To our knowledge, this is the first study illustrating vasorelaxation elicited by *Rhus coriaria*’s fruit, the part of sumac that is primarily used in traditional/folk medicine and in health-promoting diets. This is in accord with a previous investigation examining relaxation of rabbit aortic rings by sumac’s leaves (Beretta et al., 2009). It is important to note here that leaves are not the usually edible part of this plant. Hence, our results are more relevant in terms of human consumption.

Our results imply that vasorelaxation is modulated through the dual cyclic nucleotide monophosphate (cAMP and cGMP) transducing axes. Moreover, aortic relaxation to RCE is endothelium-dependent, and contingent on the signaling cascade: PI3-K/Akt, eNOS, NO, sGC, cGMP, PKG, and partaking of COX metabolites, AC and K_ATP_ channels. Conversely, the aortic relaxation is independent of muscarinic and histaminergic receptors, and voltage-gated calcium channels.

The phytochemical finger-print screening of sumac has illuminated a broad catalog of health-promoting polyphenolic components, including gallotannins and flavonoids (Beretta et al., 2009; Mehrdad et al., 2009; Regazzoni et al., 2013; Abu-Reidah et al., 2015). The most abundant botanical compounds in sumac are gallic acid (GA), and GA derivatives like the hydrolysable gallotannins, particularly pentagalloylglucose, pentagalloylglucoside, and pentagalloylhexoside (Beretta et al., 2009; Regazzoni et al., 2013; Abu-Reidah et al., 2015). In addition, the major flavonoids reported in sumac are myricetin, quercetin, kaempferol, apigenin, luteolin, rutin, and anthocyanins (red color pertaining to sumac fruits pericarp) (Mehrdad et al., 2009; Regazzoni et al., 2013; Abu-Reidah et al., 2015). These polyphenols endow sumac with properties that alleviate, prevent or resist modern-day scourges of humans, amongst which is cardiovascular disease (Heiss et al., 2010; Anwar et al., 2016; Forte et al., 2016). Contextually, the polyphenols display not only vasodilator effects, but also provide additional biological improvements associated with cardiovascular homeostasis (Loizzo et al., 2008; Beretta et al., 2009; Heiss et al., 2010; Al Disi et al., 2015; Anwar et al., 2016; Forte et al., 2016). Undoubtedly, sumac is potentially a valuable source of key phyto-therapeutic agents that are directed at multitudinal set of pathologic targets.

The endothelial cell layer is a fine orchestrator of secreted vasodilators [NO, prostacyclin (PGI_2_), and endothelium-derived hyperpolarizing factors (EDHF)] and release of vasoconstrictors [thromboxane (TxA_2_) and endothelin (ET)] (Luscher et al., 1990; Gerber et al., 1998; Triggle and Ding, 2011). Endothelial derangement ensues if there is any shift in equilibrium between these two entities, such as an aberrant NO system (Luscher et al., 1990; Hopkins, 2013). This endothelial dysfunction leads to a pathological milieu, giving rise to and exacerbating diseases such as arterial aneurysms, atherosclerosis and hypertension (Huang et al., 1995; Kuhlencordt et al., 2001; Hopkins, 2013). Therefore, pharmacological interventions to reverse vascular damage and attain homeostasis will certainly be beneficial for these pathological conditions.

Selective extracellular cues transmitted into the endothelium cause an augmented release of NO, which plays an integrative role in VSMC relaxation (Gerber et al., 1998; Anwar et al., 2013) and other physiologic and pathologic effects (Shaul, 2002; Moncada and Higgs, 2006). In the current study, relaxation was unaffected by atropine blockade. Therefore, muscarinic receptors (mAchRs), specifically those located on endothelial cell surface (Attina et al., 2008), are not involved in RCE-induced aortic relaxation. This is in agreement with previous studies using different medicinal herbs, such as *Salvia fruticosa* (Anwar et al., 2017) or *Salvia miltiorrhiza* (Lam et al., 2005), which clearly reflect on a direct herbal extract-induced vasorelaxation and hence atropine-independent effects.

Likewise, histamine-initiated vasorelaxant responses are regulated by endothelial expressed histamine H1 G protein-coupled receptors (Van de Voorde and Leusen, 1983). Again, the inhibitor pyrilamine, a blocker of histamine H1-receptors, did not alter aortic ring relaxation in our study. This result is similar to observations made on preceding studies of *Salvia fruticosa* (Anwar et al., 2017) and *Salvia miltiorrhiza* (Lam et al., 2005) on relaxation of rat isolated aortae where aortic relaxation occurred independently of histaminergic receptors.

Although not assessed in the present investigation, sumac constituents can potentially trigger a number of primary events to affect vascular reactivity. Firstly, redox-sensitive activation of PI3K/Akt/NO pathway is known to lead to arterial relaxation (Schini-Kerth et al., 2010). Secondly, phytoestrogenic polyphenols as in RCE (like quercetin, kaempferol, luteolin, and apigenin) can bind to estrogen receptors (ERs; classical: nuclear ERα and ER_β_; novel: transmembrane – G protein-coupled ER 1, GPER1/GPR30) (Prossnitz and Arterburn, 2015) to transduce NO release (Hisamoto et al., 2001; Lindsey et al., 2014; Prossnitz and Arterburn, 2015). Thirdly, inorganic nitrites and nitrates are present in RCE (Shabbir, 2012), and these anions can be metabolized to generate NO (Omar et al., 2016). These actions will benefit cardiovascular homeostasis in the disease state.

In addition to regulating cell growth, migration, proliferation, and survival (Shiojima and Walsh, 2002; Abeyrathna and Su, 2015), the PI3K/Akt plays a vital role in regulating vascular tone (Shiojima and Walsh, 2002; Abeyrathna and Su, 2015). Indeed, the PI3K/Akt pathway acts as a signaling hub for endogenous factors (Ferro et al., 2004; Olsson et al., 2006), exogenous phyto-nutrients (Anselm et al., 2007; Anwar et al., 2017), as well as prescribed medications such as pitavastatin (Wang et al., 2005). Phosphorylation of Akt leads to the stimulation of eNOS (Dimmeler et al., 1999). Activated eNOS induces the oxidation of L-arginine to L-citrulline and NO (Shaul, 2002; Moncada and Higgs, 2006). NO is widely acknowledged to be a potent rheostat of vascular tone (Gerber et al., 1998; Shaul, 2002; Moncada and Higgs, 2006). NO then activates sGC which catalyzes cGMP accumulation, which in turn activates protein kinase (PKG) in VSMCs. This PKG is known to regulate vascular tone, inhibit platelet aggregation and VSMC proliferation, and facilitates endothelial regeneration (Schlossmann et al., 2003; Kim et al., 2016). Therefore, engagement of eNOS and sGC lends credence to a previous study illustrating the relaxation of rabbit aortae with an extract of sumac leaves (Beretta et al., 2009).

Nitric oxide is the predominant mediator of vasodilation in conduit arteries, but endothelial-derived prostacyclin (PGI_2_) also contributes significantly to arterio-relaxation (Castillo et al., 1997; Gerber et al., 1998). In this context, our data reveals that pretreatment with indomethacin of aortic rings attenuated RC-induced relaxation. This result entails the participation of a COX-metabolite in response to sumac. Similarly, a preceding investigation has revealed that leaves extract of sumac caused rabbit aortic rings to relax and that this relaxation was blunted by indomethacin (Beretta et al., 2009). This study also reported that during perfusion with sumac leaves extract in ischemia-reperfusion injury of rabbit isolated heart preparations, an elevated secretion of 6-keto-prostaglandin F_1α_ was noted (Beretta et al., 2009). Endothelial-derived prostacyclin acts on the PGI_2_ receptor, coupled not only physically to heterotrimeric G proteins, but also intimately interacts with membrane-bound AC (Feletou et al., 2011). Therefore, the ability of SQ22536 to abolish RCE-induced aortic relaxation in our study is indicative of activation of AC which converts ATP to cAMP, the ubiquitous second messenger. Further, this result is reinforced by our observation of rise in cAMP release with increasing doses of sumac, and once again the generation of cAMP was antagonized by SQ22536 blockade of AC. Forskolin, a labdane diterpene isolated from tuberous roots of *Coleus forskohlii*, is known to potently activate AC and increase cAMP levels (Seamon and Daly, 1986). Importantly, a diterpenoid constituent (7 deacetyl forskolin) of sumac is also a recognized activator of AC (Zhang et al., 2009), albeit with a lower potency than forskolin in raising intracellular concentration of cAMP (Pinto et al., 2008).

Another bio-feasible way of increasing not only cAMP but also cGMP levels is through inhibition of phosphodiesterases (PDEs). These hydrolytic enzymes degrade cGMP and cAMP, and thereby terminate biological processes mediated by cyclic nucleotides. Five isoforms (PDE1 to PDE5) are expressed in the vascular wall, with PDE4 (directed at cAMP) and PDE5 (degradation of cGMP) being the most predominant isoenzymes (Bender and Beavo, 2006). Relevantly, phytochemical components of sumac fruit extract (apigenin, luteolin, myricetin, and quercetin) have been noted as inhibitors of PDEs 1-5 (Ko et al., 2004). cAMP, the prototypic second messenger, regulates diverse biological processes, amongst which are included the control of vascular tone (Garcia-Morales et al., 2017). Interestingly, cAMP modulates its actions through two distinct signaling pathways, one emanating from exchange protein directly activated by cAMP (EPAC) (Roberts and Dart, 2014) and the other arising from cAMP-dependent protein kinase (PKA) (Taylor et al., 1990). These dual cyclic AMP effectors are causative signaling factors in vascular relaxation (Shi et al., 2007; Purves et al., 2009; Garcia-Morales et al., 2017). Quercetin, a constituent of sumac, promotes PKA-dependent phosphorylation of eNOS in an Akt-independent manner (Li et al., 2012). Further, cAMP is involved in endothelium-derived relaxant effect by stimulating downstream effector molecules (PKA and EPAC), which elevate the activity of PI3K/Akt/eNOS route (Garcia-Morales et al., 2017).

In addition to playing a key role in membrane potential homeostasis, potassium channels also control vasotone in VSMCs (Nelson and Quayle, 1995). Four major classes of potassium channels are expressed in endothelial cells and VSMCs along the vascular tree, namely ATP-dependent K^+^ (K_ATP_) channels, Ca^2+^-sensitive K^+^ (K_Ca_ subtypes: BK_Ca_, IK_Ca_, and SK_Ca_) channels, inward rectifier K^+^ (Kir) channels and voltage-gated K^+^ (K_V_ subtypes: 7.4 and 7.5) channels (Nelson and Quayle, 1995; Gluais et al., 2005; Stott et al., 2016; Goto et al., 2018). The current study, using K^+^ channel inhibitors, demonstrates the involvement of K_ATP_ channels in RCE-dependent aortic relaxation, and which was not affected by a non-selective inhibitor of K_Ca_ channels. In contrast, an earlier study elaborated on the insensitivity to potassium channel inhibitor (glibenclamide) on aortic relaxant activity elicited by leaves of *Rhus coriaria* L. (Beretta et al., 2009). The differences observed between our study and a previous investigation may have arisen from the use of different animal models (rat compared to rabbit). A further explanation for the discrepancy may equate to age and gender of the animal models under investigation. The second possibility is that the concentration of individual bioactive constituents of sumac will vary according to the part of plant (leaves vs. fruit) used, in addition to the processing procedure for isolation of extract (methanolic vs. ethanolic). Another likelihood of variation may stem from absorption and metabolism of the components in the vascular wall of rats or rabbits.

The vascular K_ATP_ channel is composed of a heteromultimeric-complex of Kir6.1 (pore-forming) and SUR2B (sulfonylurea receptor) subunits (Aziz et al., 2014). Activation of K_ATP_ channels results in opening of the channel pore to cause an efflux of K^+^, leading to membrane hyperpolarization, with a concurrent decrease in Ca^2+^ entry, and onto vasodilation (Nelson and Quayle, 1995). Vascular smooth muscle Kir6.1 subunit of K_ATP_ channels contributes to regulation of vascular tone, and hence blood pressure homeostasis. This action is irrespective of gender (Aziz et al., 2014). Consistent with our finding is that K_ATP_ channels are activated by vasodilators coupled to PKA (Quinn et al., 2004). Protein kinase A modulates the activation of K_ATP_ by phosphorylating Ser-385 in Kir6.1 subunit, and Thr-633 and Ser-1465 on SUR2B subunit to proceed onto activation (Quinn et al., 2004). In a subsequent study, a group of investigators revealed that vasorelaxant activity is also evoked by PKA phosphorylation of SUR2B subunit at Ser-1387 to modulate K_ATP_ channel events (Shi et al., 2007). Unlike PKA, the other effector of cAMP, EPAC, appears to inhibit the same downstream target, the K_ATP_ channels (Purves et al., 2009). This adds to the complexity of this system; however, it may be explained by cAMP camps. Indeed, AC was reported to reside in caveolae microdomains (Huang et al., 1997; Rybin et al., 2000; Ostrom et al., 2001; Smith et al., 2002; Bundey and Insel, 2004). Moreover, “local cAMP signals” have also been alluded to, with the interesting notion that restriction of cAMP signaling within microdomains plays a role in selective or preferential activation of certain signaling pathways (Schwencke et al., 1999; Cooper, 2003).

In terms of the NO/cGMP mechanistic pathway, PKG is known to stimulate K_ATP_ channels (Nelson and Quayle, 1995), as may occur in RCE-induced vasorelaxation. Thus, both PKA and PKG may act synergistically (signaling convergence) to augment RCE-initiated vasodilatation; as previously elucidated with albuterol, an agonist for β_2_-adrenergic receptors, as well as stimulation of K_ATP_ channels (Ferro et al., 2004; Quinn et al., 2004). It is important to note that during the preparation of this manuscript, a study characterizing the molecular and functional aspects of an endothelial ATP-sensitive potassium channel was published (Aziz et al., 2017). Using various techniques such as patch-clamp, RT-PCR, calcium imaging as well as *ex vivo* coronary perfusion Langendorff heart experiments, this paper elegantly and conclusively showed that the vascular endothelium does indeed express functional Kir6.1-containing K_ATP_ channels. As such, the role of these channels cannot be eliminated, and thus remains to be established in RCE-exerted relaxation.

Voltage-dependent potassium channels have not been examined in the present study, but it is feasible that RCE-mediated relaxation is modulated by both cAMP and cGMP pathways via stimulation of K_V_ 7.4/7.5 channels (Stott and Greenwood, 2015). Moreover, EPAC-dependent arterial relaxation can occur in part via activation of K_V_ 7.4 channels (Stott and Greenwood, 2015). Importantly, EPAC is also known to raise the activity of BK_Ca_ channels leading to VSM relaxation (Roberts et al., 2013).

The present data illustrates that vasorelaxation is independent of extracellular calcium influx as verapamil (L-type Ca^2+^ channel blocker) failed to modify RCE-driven relaxations of aortic rings. This result is similar to a recent report where L-type Ca^2+^ channel blockers did not affect extract-induced aortic relaxation (Anwar et al., 2017).

Endothelium-independent relaxation can be explained by a number of mechanisms targeting the VSM, including the direct stimulation of AC. Forskolin, a labdane diterpene isolated from tuberous roots of *Coleus forskohlii*, is known to potently activate AC and increase cAMP levels (Seamon and Daly, 1986). Importantly, a diterpenoid constituent (7 deacetyl forskolin) of sumac is also a recognized activator of AC (Zhang et al., 2009), albeit with a lower potency than forskolin in raising intracellular concentration of cAMP (Pinto et al., 2008). Moreover, a recent study has elucidated the interactions between quercetin, a flavonoidal constituent of RCE, and voltage-gated potassium channels (K_V_), causing hyperpolarization, through an increase in K^+^ efflux, of rat coronary arterial smooth muscle cells (Hou et al., 2014). The contribution of the above-mentioned factors will be explored in future studies.

## Conclusion, Limitations, and Future Perspectives

**FIGURE 9:**
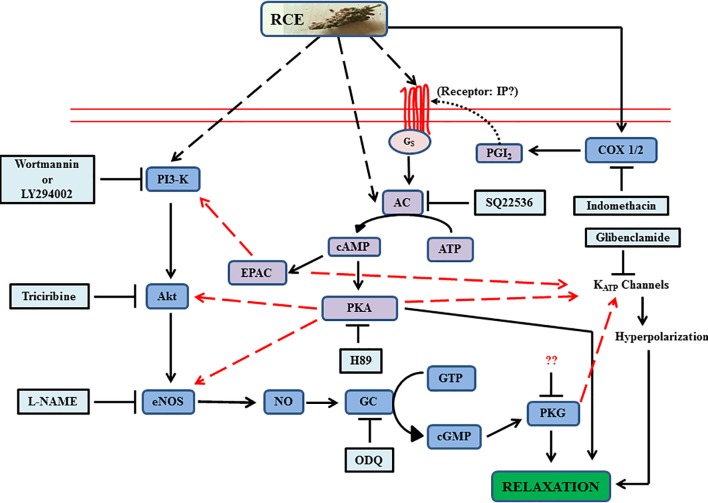
Summary of experimental data of *Rhus coriaria* (sumac)-evoked signaling in aortic relaxation. The diagram illustrates guanylyl cyclase’s (royal blue color) role as a nexus between the proximal activators and distal effectors, and also incorporating the activation of adenylyl cyclase system (purple). Additional contributory routes are implicated by red dashed arrows (convergence hubs for cross-talk between the two systems). Light blue boxes are antagonists. RCE, *Rhus coriaria* extract; AC, adenylyl cyclase; EPAC, exchange protein directly activated by cAMP; NO, nitric oxide; eNOS, endothelial nitric oxide synthase. Please see text for details. Solid lines in the figure are either established in this study or already published.

To summarize, our data has emphasized the significant value of sumac fruit in inducing beneficial vasorelaxant actions through AC and GC signaling pathways, including the potential of cross-talk between the two systems (**Figure 9**). Collectively, the analysis of the data supports and offers a mechanistic explanation for the use of sumac in folk medicine for amelioration of cardiovascular diseases. To consolidate on the present information, our next step will be to advance the proof of concept by exploring the effects of sumac on atherosclerosis, aortic aneurysms and hypertension (macro- and micro-vascular diseases) in preclinical models, and if successful to go on to translate this into clinical practice. These diseases, causes of disability and death worldwide, are certainly deemed to be preventable by adherence to healthy lifestyle factors, particularly the diet as pointed out in the introduction.

*Rhus coriaria*’s fruit is endowed with a rich array of phytomedicinal compounds. From the preceding account, it is reasonable to assume that an extract of sumac fruit (varied complement of standardized bioactive constituents) is probably functionally more advantageous as a therapeutic platform for targeting multiple pathological pathways rather than any individual phytochemical. These botanical-constituents do not pose any health-risks, and will be very cost-effective compared to the currently available anti-atherogenic and anti-hypertensive prescribed medication.

Despite the strong evidence this paper adds to the already established benefits of consuming sumac for the management and treatment of several ailments, our study is not without some limitations. These limitations are common to other herbs/plants utilized in herbal medicine. Some of these limitations include seasonal variations in the bioactive content of the herbal part; this may be overcome by detailed characterization and standardizing of the plant extract to be used. Other factors to consider are the variety, regional differences in soil and climate, age/maturity, cultivation of *Rhus coriaria* at the time of harvest. Additional limitations may include lack of sufficient awareness among patients and physician regarding the abuse or use of plant-derived products, misclassification, misidentification or adulteration of medications based on herbal prescriptions. Perhaps, then, involving governmental or health-related bodies in overseeing the production and standardization of such herbal or plant-inspired products may alleviate the above limitations.

## Author Contributions

AE conceived of the project. AE and RI designed the experiments. MA helped in the design of some experiments. MA and AE performed the data analysis and wrote the manuscript. AS and SB performed the experiments. All authors read and approved the submitted version.

## Conflict of Interest Statement

The authors declare that the research was conducted in the absence of any commercial or financial relationships that could be construed as a potential conflict of interest.

## Supplementary Material

FIGURE S1 Endothelium-intact rings were treated with cumulative doses of sumac in the absence (RCE; circles) or presence (LY + L-NAME + RCE; squares) of combined LY294002 (10 μM) and L-NAME (100 μM). Values are mean ± SEM, *p* < 0.05.

FIGURE S2 Endothelium-intact rings were treated with cumulative doses of sumac in the absence (RCE; circles) or presence (LY + L-NAME + RCE; squares) of combined LY294002 (10 μM) and SQ22435 (10010 μM). Values are mean ± SEM, *p* < 0.05.
