# Supplementary figures and images for: Rhus coriaria L. (Sumac) Evokes Endothelium-Dependent Vasorelaxation of Rat Aorta: Involvement of the cAMP and cGMP Pathways
Source: Front Pharmacol. 2018 Jun 28;9:688. doi: 10.3389/fphar.2018.00688 (PMC6031713; doi:10.3389/fphar.2018.00688)

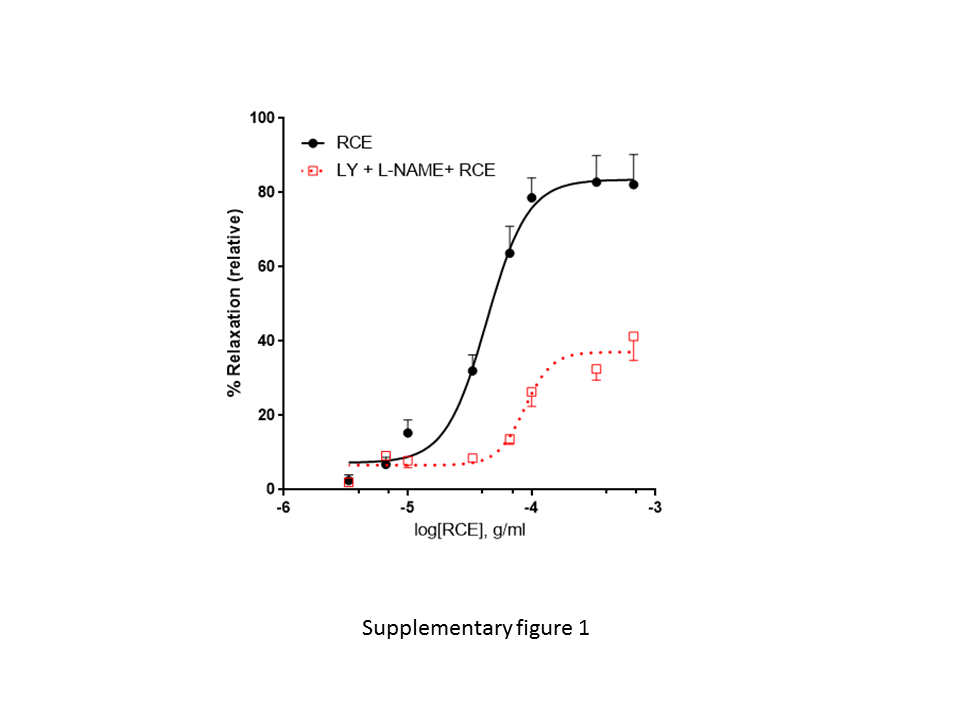

Supplement: FIGURE S1 — Endothelium-intact rings were treated with cumulative doses of sumac in the absence (RCE; circles) or presence (LY + L-NAME + RCE; squares) of combined LY294002 (10 μM) and L-NAME (100 μM). Values are mean ± SEM, p < 0.05. [file Image_1.TIF]

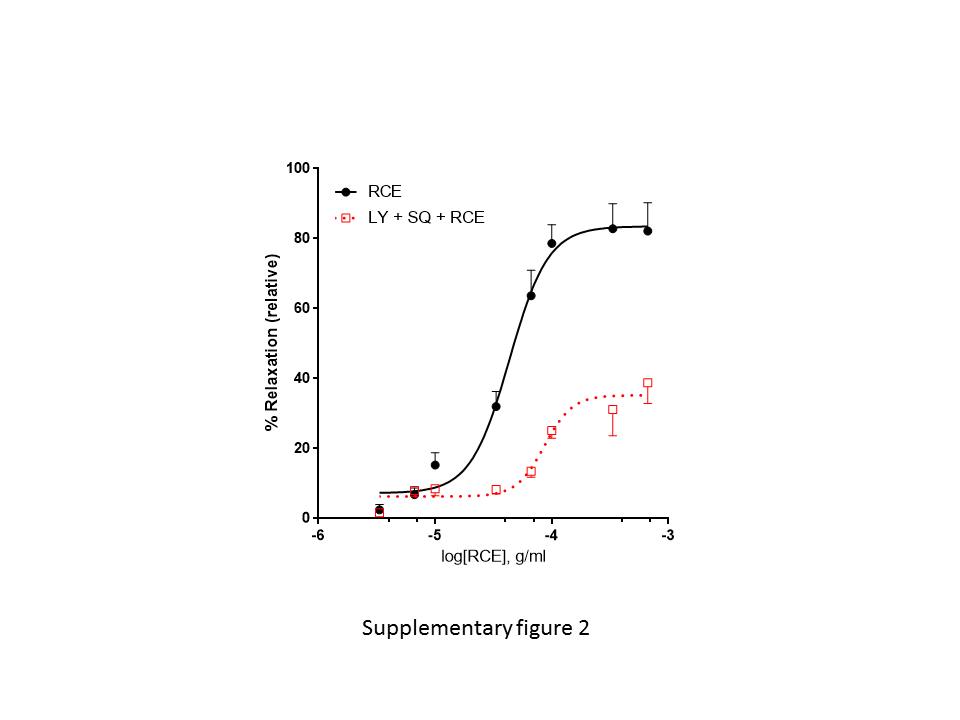

Supplement: FIGURE S2 — Endothelium-intact rings were treated with cumulative doses of sumac in the absence (RCE; circles) or presence (LY + L-NAME + RCE; squares) of combined LY294002 (10 μM) and SQ22435 (10010 μM). Values are mean ± SEM, p < 0.05. [file Image_2.TIF]
